# Technique for Measuring Limb Occlusion Pressure that Facilitates Personalized Tourniquet Systems: A Randomized Trial

**DOI:** 10.1007/s40846-016-0173-5

**Published:** 2016-10-04

**Authors:** Bassam A. Masri, Brian Day, Alastair S. E. Younger, Jeswin Jeyasurya

**Affiliations:** 1 Department of Orthopaedics, University of British Columbia, 3114 - 910 West 10th Avenue, Vancouver, BC V5Z 1M9 Canada; 2 Department of Orthopaedics, University of British Columbia, 560 1144 Burrard Street, Vancouver, BC V6Z 2A5 Canada; 3 Western Clinical Engineering, 207-1099 West 8th Avenue, Vancouver, BC V6H 1C3 Canada

**Keywords:** Tourniquet, Limb occlusion pressure, Personalized, Tourniquet safety, Tourniquet injury

## Abstract

We developed a technique for measuring patient limb occlusion pressure (LOP) through a tourniquet cuff that overcomes many limitations of existing LOP measurement techniques. The purpose of the study is to determine whether the LOP measured by the proposed technique is statistically or clinically different from that measured by the gold standard Doppler ultrasound technique. The study used randomized crossover multicenter trials. 143 pre- and post-surgical patients with a mean age of 54 years (range 17–86 years) were enrolled in the study. Pneumatic cuffs were applied to the non-operative upper and lower limbs and LOP was measured using the proposed technique and the Doppler ultrasound technique. From a total of 252 usable measurements for each technique (134 for upper limbs and 118 for lower limbs), the mean difference in LOP between the two techniques was 1 ± 8 mmHg for the upper limbs, 0 ± 15 mmHg for the lower limbs, and 1 ± 12 mmHg overall. The differences between the proposed technique and the Doppler technique were neither statistically nor clinically significant. The simplicity, effectiveness, and accuracy of the proposed technique should lead to broader clinical usage and acceptance of LOP measurement, thus leading to safer, personalized pressures in surgical tourniquet applications.

## Introduction

Advances in pneumatic tourniquet technology over the last 35 years have significantly improved the safety, efficacy, and reliability of tourniquet instruments and cuffs. However, tourniquet-related nerve injury is a potentially harmful complication of tourniquet use. Nerve injuries from tourniquet use range from a mild transient loss of function to permanent, irreversible damage [1]. Ochoa et al. [2–4] showed that in most cases, nerve damage is limited to the part of the nerve that is underneath and near the edges of the cuff and that the underlying cause of tourniquet paralysis is compressive neurapraxia rather than ischemic neuropathy or muscle damage. Compression of the large myelinated fibers underneath the tourniquet cuff results in displacement of the node of Ranvier from its usual position under the Schwann-cell junction. Studies of the distribution of pressures beneath tourniquet cuffs have shown that high tourniquet inflation pressures in narrow uncontoured tourniquet cuffs result in high pressure gradients near the cuff edges. In turn, this results in higher compressive pressures and higher pressure gradients along the underlying nerves and soft tissues [5, 6]. Consequently, higher levels of tourniquet inflation pressure and higher pressure gradients beneath tourniquet cuffs are associated with a higher risk of nerve-related injury [1].

Specific advances in tourniquet technology have reduced the risk of nerve-related injury in recent years. These advances include the design of tourniquet cuffs with lower pressure gradients, and the use of Limb Occlusion Pressure (LOP) to set optimal personalized tourniquet pressure, rather than setting standard tourniquet inflation pressures, which are typically higher and more hazardous. LOP is defined as the minimum pressure required, at a specific time in a specific type of tourniquet cuff applied to a specific patient’s limb at a specific location, to stop the flow of arterial blood into the limb distal to the cuff [1]. However, the adoption of personalized tourniquet settings based on LOP has been limited by practical difficulties of LOP determination. These limitations include the need for a distal blood flow sensor, which adds cost and complexity, and affects the preparation of the sterile field; the effect on perioperative workflow and time; and the success rate of LOP measurement being dependent on variables affecting the measurement of low peripheral blood flow [7].

An automatic technique for measuring LOP was developed in the present study in an effort to overcome these limitations. It uses a tourniquet cuff with a continuous pneumatic passageway surrounding the limb as a dual-purpose patient sensor and pneumatic effector. This circumvents the need for a distal sensor, a limiting factor in prior adoption of LOP measurement. If this technique is shown to have accuracy comparable to that of the current gold standard technique of LOP measurement [8], its acceptance should lead to broader clinical usage of LOP measurement, thus leading to safer personalized pressures in surgical tourniquet applications.

The purpose of this study is to determine whether the LOP measured by the proposed technique is statistically or clinically different from that measured by the gold standard Doppler technique [8] using a randomized crossover multicenter trial.

## Materials and Methods

### Participants

The experiment was conducted in three surgical centers in Vancouver, British Columbia, namely the Cambie Surgery Centre, the Complex Joint Clinic at Vancouver General Hospital, and the Foot and Ankle Clinic, St. Paul’s Hospital. Recruitment and data collection commenced in September 2014 and finished in December 2014. Participants were recruited either by a mailed letter sent to them prior to their clinic visit, or during their clinic visit, where they were asked to participate by an assigned research assistant or clinic staff. All participants gave their informed consent prior to their inclusion in the study.

The study enrolled 143 pre- and post-surgical patients aged 17–86 years (mean ± SD, SD: 54 ± 15 years) in three surgical clinics located in Vancouver, British Columbia, Canada: Cambie Surgery Centre (80 patients); Complex Joint Clinic, Vancouver General Hospital (52 patients); Foot and Ankle Clinic, St. Paul’s Hospital (11 patients). Table 1 lists the inclusion and exclusion criteria. Participants were enrolled by clinic staff and two experimenters. Subjects were asked whether they met any exclusion criteria and were excluded from the trial if they had answered yes to one or more of the exclusion criteria. Table 2 provides a summary of the patient demographic data. Randomization of limb and measurement order was completed using a computerized random number generator to create a numbered list of random allocation sequences. Participants were assigned in consecutive order to the randomized sequences on the list. The two experimenters generated the random allocation sequence for the LOP measurements, assigned participants to the LOP measurements, and completed the measurements. The research was approved by the University of British Columbia Research Ethics Board (certificate H14-02048) and was therefore performed in accordance with the ethical standards laid down in the 1964 Declaration of Helsinki.

**Table 1:** Inclusion and exclusion criteria

Inclusion criteria
Scheduled for a visit to one of three surgical clinics in Vancouver, Canada
Agreed to participate in the study and provided informed consent
Exclusion criteria
Unable to give informed consent on their own behalf
Standard contraindications to tourniquet use
Vascular disease or circulation problems in the extremities
History or indication of deep vein thrombosis

**Table 2:** Summary of anthropometric data from 143 pre- and post-surgical adult patients (47 female, 96 male)

	Age (yr)	Mass (kg)	Height (cm)	Upper-limb circ. (cm)	Lower-limb circ. (cm)
Mean ± SD	54 ± 15	83 ± 17	173 ± 9	32 ± 3	55 ± 5

### Experimental Design

#### Proposed Tourniquet Design

The proposed technique for measuring LOP involves the use of unique dual-purpose disposable tourniquet cuffs along with a tourniquet instrument containing LOP calculation sensors and software. The tourniquet cuffs surround and conform closely to a range of underlying limb shapes, and have a stiffened two-layer design that incorporates a continuous pneumatic passageway that completely surrounds the underlying limb after application. These cuffs can be used for LOP measurement or as a tourniquet to safely stop arterial blood flow during a surgical procedure. The cuffs are described in US Patent 8425,551.

**Fig. 1:**
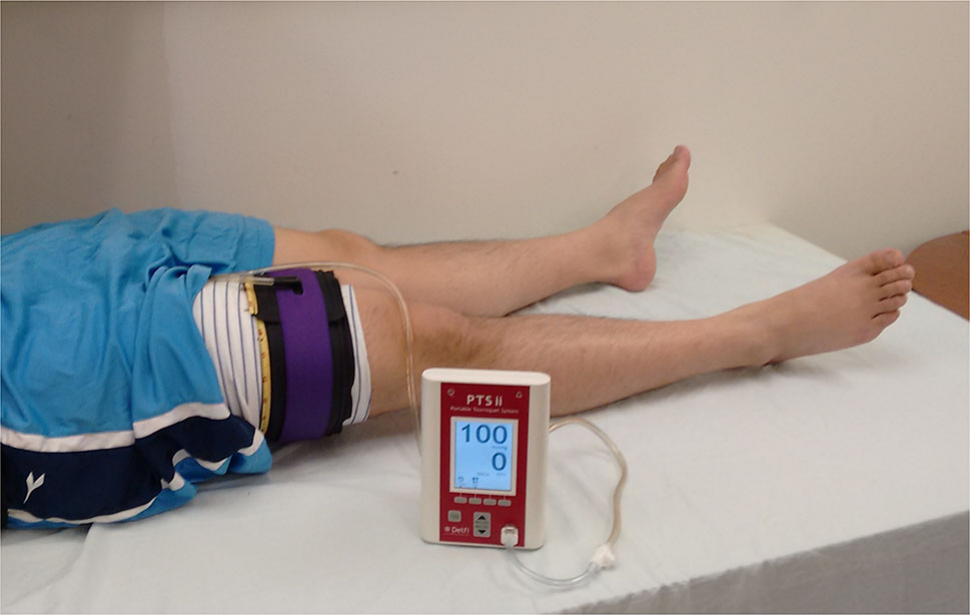
Photograph of a volunteer with dual-purpose two-layer cuff and matching limb protection sleeve applied to limb. Cuff is pneumatically connected to tourniquet instrument that contains LOP calculation sensors and software

The instrument connected to the tourniquet cuff increases the cuff pressure in 10-mmHg stepwise increments, analyzes the pneumatic pressure pulsations induced in the cuff bladder by the arterial pressure pulsations at each cuff pressure increment, and uses these characteristics to determine LOP. Figure 1 shows the instrument and cuff applied to a lower limb.

#### Experimental Procedure

Each patient was asked to lie on a clinic bed and an appropriately sized dual-purpose tourniquet cuff with an underlying matching limb protection sleeve was applied to the non-surgical upper arm and non-surgical thigh, in sequence, by an experimenter. The upper and lower limb tourniquet cuffs were not inflated at the same time. A standard blood pressure cuff was applied to the other arm.

The patient was instructed to inform the experimenter if the cuff pressure became uncomfortable and measurements were discontinued if a patient requested the cuff to be deflated prior to completion of the measurement.

The patient’s blood pressure was taken at the start and end of the measurement sequence. LOP measurements were taken on the non-operative upper and lower limbs. On each limb, one LOP measurement was taken using the proposed technique (experimental) and one measurement was taken using the Doppler technique (gold standard/control). The limit for maximum applied tourniquet pressure was 340 mmHg and measurements were taken in randomized order (either upper or lower limb first; and either proposed or Doppler technique first), with randomization determined using a computerized random number generator.

For upper-limb measurements using the Doppler technique, one experimenter positioned the Doppler probe at the radial artery in the wrist to monitor arterial flow distal to the tourniquet. For lower-limb measurements, this experimenter positioned the Doppler probe at either the dorsalis pedis artery or the posterior tibial artery on the foot to monitor arterial flow distal to the tourniquet. Once the first experimenter had positioned the Doppler probe and heard a clear distal pulse, a second experimenter continuously inflated the tourniquet cuff using the manual pressure regulator up to the pressure at which the first experimenter verbally indicated that he could no longer hear the distal pulse. This pressure was recorded by the second experimenter as the estimated LOP. The first experimenter was blinded to the cuff pressure during both types of measurement. Both experimenters took turns operating the Doppler probe to reduce experimenter-based measurement bias.

For the proposed technique, the instrument increased the cuff pressure in 10-mmHg stepwise increments, analyzed the pneumatic pressure pulsations induced in the cuff bladder by the arterial pressure pulsations at each cuff pressure increment, and used characteristics of the pulsations as the cuff pressure was incremented to determine LOP. The LOP measured by the proposed technique was recorded by the second experimenter to ensure that the first experimenter (operating the Doppler probe) was blinded to this measurement.

In both techniques, the cuff was immediately deflated after LOP was determined. Measurements discontinued due to patient-specific factors or due to a data collection error were excluded from the analysis. The reasons for exclusion were documented and are listed in Table 3 for each trial.

**Table 3:** Data excluded from analysis

	Upper limb	Lower limb
Patient-specific factor		
Patient left for surgery before data collection started	1	1
Discomfort leading to discontinued measurement	0	9
LOP higher than permitted by protocol	0	10
Existing nerve sensitivity to higher cuff pressures	0	2
Patient movement causing loss of Doppler signal	1	0
Data collection error		
Tourniquet instrument error during measurement	2	0
Data collection software saving error	5	3
Total	9	25

#### Statistical Analysis

The variable studied was the LOP measured, in mmHg, using the Doppler technique and proposed automatic technique. To determine the accuracy of the proposed technique of LOP measurement (experimental) compared to that of the Doppler technique (gold standard/control), data were analyzed for differences between each pair of LOP measurements. The mean of the LOP differences for each limb were calculated, as well as the ranges, SDs, standard errors, and 95 % confidence intervals of the means. Differences in measurements were compared using a paired *t* test. Statistical significance was defined as a *p*-value of less than 0.05.

The distribution of the differences was plotted using a histogram and a Bland–Altman plot [9]. The Bland–Altman plot also shows the mean and two SDs of the differences. Following the method described by Bland and Altman [9], measurement pairs with LOP differences of greater than two SDs from the mean were defined as outliers. Additional analysis was completed on the recorded pressure pulsation data in these measurements to identify any specific pulsation characteristics that were unique to outliers. Statistical analysis of the LOP differences was repeated after the removal of the outliers.

An initial sample size of 100 patients was defined after examining the sample sizes of two similar studies (both used 20 patients) [10, 11]. We decided to select a larger sample size to increase the statistical confidence of our results.

## Results

**Fig. 2:**
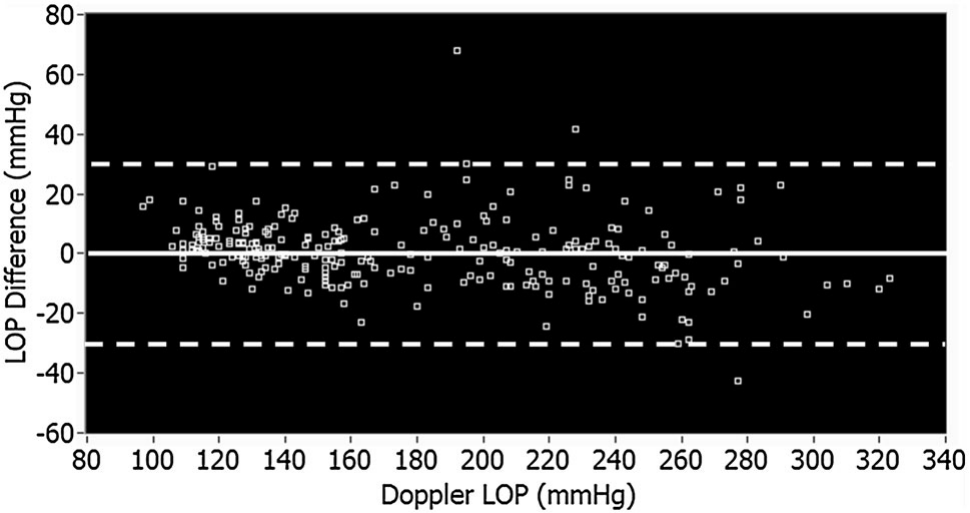
Bland–Altman Plot of LOP difference between proposed technique and manual Doppler technique for all limbs. Mean difference is shown (*solid line*) plus or minus two standard deviations (*dashed lines*)

**Fig. 3:**
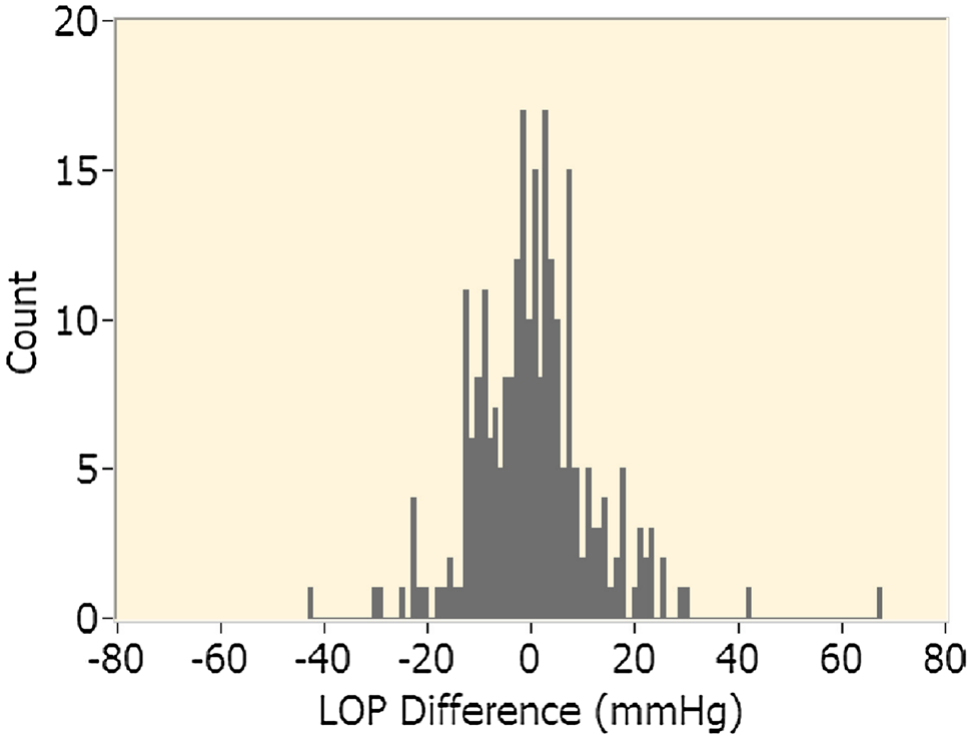
Histogram of LOP difference between proposed technique and manual Doppler technique for all limbs

From the 143 patients enrolled in the study, usable data were collected from 252 limbs (134 upper limbs and 118 lower limbs). Data collection continued beyond the sample size limit because data collection and patient consent were both scheduled ahead of time in two surgical clinics (Complex Join Clinic and Foot and Ankle Clinic). Data collection stopped after the scheduled clinic days were completed. Table 3 contains a list of factors leading to data collection errors or exclusions. LOP difference was defined as the LOP obtained using the proposed technique minus that obtained using the Doppler technique. As such, a positive difference indicates a higher reading for the proposed technique. The *p* values, ranges, means, SDs, standard errors, and 95 % confidence intervals of the means of the LOP differences between the proposed technique and the Doppler technique are presented in Table 4. The LOP differences for all measurements are shown in Fig. 2 and a histogram of the LOP differences is shown in Fig. 3.

**Table 4:** LOP difference (proposed-Doppler)

Limb	No. of limbs	Min diff. (mmHg)	Max diff. (mmHg)	Mean diff. (mmHg, mean ± SD)	SE of mean (mmHg)	95 % confidence interval of difference (mmHg)		Paired *t*-test
						Lower	Upper	*p* value
Upper	134	−23	29	1 ± 8	1	−1	3	0.14
Lower	118	−43	68	0 ± 15	1	−3	3	0.95
Combined	252	−43	68	1 ± 12	1	−1	2	0.45

In one LOP measurement using the proposed technique, significant fluctuations in pressure pulsation data indicative of pneumatic noise were observed, indicating a future need for better signal filtering and automatic noise detection and reduction. This patient’s data were included in the statistical analysis.

The means and SDs listed in Table 4 were used to compare the accuracy of the proposed technique with the Doppler technique. The *p* values were greater than 0.05 for all of the paired *t*-tests, showing that there is no statistically significant difference between the two measurement methods. The SD of the lower-limb LOP difference was higher than that of the upper-limb LOP difference (15 mmHg). This SD is not clinically significant. An absolute LOP difference of greater than two SDs for the lower-limb mean LOP difference was defined as an outlier measurement (>30 mmHg). Three lower-limb LOP measurements using the proposed technique had LOP differences of greater than 30 mmHg and were classified as outliers. There were no outliers in the upper-limb measurements.

In further analysis of the three outliers, a predictable difference was identified in the sets of pulsation characteristics at corresponding cuff pressure increments when compared to those for non-outlier data. This difference could form the basis for adding rules to the algorithm of the proposed LOP measurement technique, allowing automatic identification of outlier subject data prior to the completion of LOP measurements, and thus allowing the surgeon to over-ride the proposed tourniquet protocol and revert to a Doppler LOP measurement if desired.

Table 5 shows the results of the data with the outlier data from the three patients removed by application of these rules: the SD of the lower-limb measurement is reduced by 2 mmHg.

**Table 5:** LOP difference (proposed-Doppler) with outliers removed

Limb	No. of limbs	Min diff. (mmHg)	Max diff. (mmHg)	Mean diff. (mmHg, mean ± SD)	SE of mean (mmHg)	95 % confidence interval of difference (mmHg)		Paired *t*-test
						Lower	Upper	*p* value
Upper	134	−23	29	1 ± 8	1	−1	3	0.14
Lower	115	−30	30	−1 ± 13	1	−3	2	0.68
Combined	249	−30	30	0 ± 10	1	−1	2	0.65

Of the 34 measurement pairs excluded from the analysis for reasons listed in Table 3, one exclusion was due to a Doppler measurement error and two exclusions were due to an instrument error during the measurement using the proposed technique.

## Discussion

Tourniquet-related nerve injury is a potentially harmful complication of tourniquet use with injuries ranging from a mild transient loss of function to permanent, irreversible damage. Evidence shows that higher tourniquet pressures are associated with higher probabilities of injury [1]. Advances in tourniquet technology have resulted in the use of lower tourniquet pressures; however, the use of personalized tourniquet settings based on LOP has been limited by practical difficulties of manual LOP determination using Doppler ultrasound, and because of limitations inherent in the distal-sensor-based technique of automatic LOP measurement [7].

Our study has some limitations. As in all studies of this nature, the size of the study population is necessarily small compared to the overall patient population. Another limitation was the inability to repeat measurements to verify the repeatability of measurements. The clinical settings in which the study was conducted prevented repeated LOP measurements on patients due to time constraints and potential patient discomfort and anxiety. The narrow 95 % confidence interval of the difference and small standard error of the mean show that these limitations do not jeopardize our conclusions.

The LOP measured using the proposed technique is not clinically nor statistically different from that measured using the gold standard (Doppler ultrasound).

Several studies have investigated the measurement of LOP for determining personalized tourniquet pressure levels and reducing applied tourniquet pressures and pressure gradients. Graham et al. [8] described the need for lowering tourniquet pressure due to shear forces in the limb from tourniquet pressure that can result in structural damage to underlying nerves. They used a Doppler ultrasound LOP measurement technique to show that wide tourniquet cuffs have lower LOPs than those of narrow tourniquet cuffs. A study by Younger et al. [11] investigating wide tourniquet cuffs and the distal-sensor-based automatic LOP measurement technique demonstrated that this technique used in conjunction with wide cuffs significantly lowered the tourniquet pressure required to occlude blood flow without compromising the quality of the surgical field. This result was confirmed in a similar study on pediatric patients by Reilly et al. [12]. McEwen et al. [10] compared the accuracy of the distal-sensor-based technique of automatic LOP measurement with that of the Doppler ultrasound technique and showed that the former has a surgically acceptable level of accuracy. 39 pairs of measurements were taken on the lower limbs of 20 healthy adult subjects and the mean difference between the two techniques was 1.7 ± 8.9 mmHg (mean ± SD).

The study by McEwen et al. [10] comparing the distal-sensor-based automatic technique of LOP measurement with the Doppler technique in the lower limbs of healthy subjects had an SD of 8.9 mmHg in the mean difference between the two techniques. This is slightly lower than the 13-mmHg SD for the lower limbs found in our study after the removal of outliers. However, the sample size of 39 measurement pairs in that study was much lower than the 118 lower-limb measurement pairs in our study.

These initial results demonstrate that the proposed technique of LOP measurement has surgically acceptable accuracy that is comparable to that of Doppler ultrasound, and that the proposed technique is feasible for incorporation into improved personalized tourniquet systems. The accuracy is comparable to that of the distal-sensor-based automatic method of LOP measurement, as determined by McEwen et al. [10]. Further, many limitations of the present techniques of LOP measurement are overcome with the proposed technique. Examples include: a distal blood flow sensor is no longer required; the sterile field is unaffected; perioperative workflow and time are less affected as this technique allows measurement of the LOP while the limb is elevated and being prepared for surgery; and the success rate of LOP measurement should be substantially greater because the proposed technique is not dependent on variables affecting the measurement of low blood flow distal to the cuff, such as cold digits or poor peripheral circulation.

The results of this study can be used to develop personalized tourniquet systems consisting of unique dual-purpose cuffs connected to instruments suitable for measuring tourniquet LOP with the proposed measurement technique. The simplicity, effectiveness, and accuracy of this technique should lead to broader clinical usage and acceptance of LOP measurement, thus leading to safer, personalized pressures in surgical tourniquet applications.
